# Identification of biomarkers for periodontal disease using the immunoproteomics approach

**DOI:** 10.7717/peerj.2327

**Published:** 2016-08-24

**Authors:** Jesinda P. Kerishnan, Sani Mohammad, Muhamad Shaifunizam Alias, Alan Kang-Wai Mu, Rathna Devi Vaithilingam, Nor Adinar Baharuddin, Syarida H. Safii, Zainal Ariff Abdul Rahman, Yu Nieng Chen, Yeng Chen

**Affiliations:** 1 Department of Oral Science & Craniofacial, Faculty of Dentistry, University of Malaya, Kuala Lumpur, Malaysia; 2 Faculty of Dentistry, University of Malaya, Kuala Lumpur, Malaysia; 3 Laboratory of Biotechnology, Institute of Win Men Biotech, Sungai Bakap, Pulau Pinang, Malaysia; 4 Department of Restorative Dentistry, Faculty of Dentistry, University of Malaya, Kuala Lumpur, Malaysia; 5 Department of Oro-Maxillofacial Surgical & Medical Sciences, Faculty of Dentistry, University of Malaya, Kuala Lumpur, Malaysia; 6 Chen Dental Specialist Clinic, Kuching, Sarawak, Malaysia; 7 Oral Cancer Research and Coordinating Centre, Faculty of Dentistry, University of Malaya, Kuala Lumpur, Malaysia

**Keywords:** Immunoblotting, Biomarker, Proteomics, Periodontal disease, Serum immunogenic protein, Two-dimensional electrophoresis

## Abstract

**Background:**

Periodontitis is one of the most common oral diseases associated with the host’s immune response against periodontopathogenic infection. Failure to accurately diagnose the stage of periodontitis has limited the ability to predict disease status. Therefore, we aimed to look for reliable diagnostic markers for detection or differentiation of early stage periodontitis using the immunoprotemic approach.

**Method:**

In the present study, patient serum samples from four distinct stages of periodontitis (i.e., mild chronic, moderate chronic, severe chronic, and aggressive) and healthy controls were subjected to two-dimensional gel electrophoresis (2-DE), followed by silver staining. Notably, we consistently identified 14 protein clusters in the sera of patients and normal controls.

**Results:**

Overall, we found that protein levels were comparable between patients and controls, with the exception of the clusters corresponding to A1AT, HP, IGKC and KNG1 (p < 0.05). In addition, the immunogenicity of these proteins was analysed via immunoblotting, which revealed differential profiles for periodontal disease and controls. For this reason, IgM obtained from severe chronic periodontitis (CP) sera could be employed as a suitable autoantibody for the detection of periodontitis.

**Discussion:**

Taken together, the present study suggests that differentially expressed host immune response proteins could be used as potential biomarkers for screening periodontitis. Future studies exploring the diagnostic potential of such factors are warranted.

## Introduction

Periodontitis, a chronic inflammatory condition in periodontal supporting structures, is the most common oral inflammatory disease and presents a dental biofilm as its primary etiology (Löe, Theilade & Jensen, 1965). A harmonious balance between the microbiota present in the dental biofilm and host immunity is a sign of periodontal health. However, when this relationship is disturbed by an over stimulated or diminished immune response toward periodontopathogens, periodontitis develops and may ultimately lead to tooth loss (Bartold & Van Dyke, 2013).

There are two major forms of periodontitis, chronic periodontitis (CP) and aggressive periodontitis (AP) (Armitage, 1999). CP is a slowly progressing disease, which can be categorized as mild/early, moderate or severe based on clinical criteria, such as probing pocket depth (PPD) and clinical attachment loss (CAL) (Armitage, 1999; Eke et al., 2012; Wiebe & Putnins, 2000). CP has a high prevalence, with approximately 40% of patients suffering from moderate forms and 8% diagnosed with severe forms (Albandar, Brunelle & Kingman, 1999; Eke et al., 2012). In contrast, AP differs from CP based on the rapid rate of disease progression in an otherwise clinically healthy patient, absence of large accumulations of plaque/calculus, and familial aggregation of diseased individuals (Armitage, 1999). AP is known to occur in 1–3% of the population (Susin, Haas & Albandar, 2014).

Traditional diagnostic procedures (e.g., visual examination, tactile sensation, PPD, CAL, plaque index, bleeding on probing, and radiographic assessment of alveolar bone loss) are still currently practised and considered to be fundamental for diagnosis (Hatem, 2012). That being said, the standard protocol for measuring CAL and PPD using a manual probe was first described more than five decades ago, and very little has changed since 1959 (Kinney, Ramseier & Giannobile, 2007). Although, such procedures might measure accumulated previous disease at a specific site (Ramfjord, 1959), they lack accuracy and are unable to predict ongoing disease activity (Chapple, 1997). For this reason, there remains a fundamental need to develop accurate, precise, and reproducible biomarkers to diagnose periodontal disease, similar to those used in medical disciplines (Preshaw, 2009).

Immunoglobulin M (IgM) is a natural antibody that can bind to specific antigens to which the host has never been exposed (Avrameas, 1991; Casali & Schettino, 1996; Coutinho, Kazatchkine & Avrameas, 1995; Pereira et al., 1986). This natural immunoglobulin exhibits traits that permit it to bind to antigens as they invade, resulting in complement activation as a first line defence mechanism. Moreover, IgM displays multi-valency and naturally high avidities (Coutinho, Kazatchkine & Avrameas, 1995; Fearon & Locksley, 1996; Tlaskalová-Hogenová et al., 1991). Therefore, low-level antigenic or immunogenic proteins are known to drive IgM-induced immune responses (Lin et al., 2007). In this regard, the participation of IgM in the early recognition of bacteria in periodontal disease has been demonstrated (Vollmers & Brändlein, 2006).

Immunoproteomics is the large-scale study of proteins (proteomics) involved in the immune response. A wide variety of potential periodontal proteomic markers are included within the immunoproteome, from immunoglobulins to bone remodelling proteins. For this reason, the use of immunoproteomic approaches in combination with current clinical examination tools could lead to the development of more reliable and effective diagnostic methods to differentiate AP and CP patients. Therefore, this study aims to use two-dimensional gel electrophoresis (2-DE) and immunoblotting approaches to identify immunogenic proteins with diagnostic potential in sera from patients with periodontitis.

## Materials and Methods

### Clinical samples

Serum samples were obtained from the Malaysian Periodontal Database and Biobank System (MPDBS) (Vaithilingam et al., 2015) (University of Malaya, Kuala Lumpur, Malaysia) with approval from the Ethics Committee of the Faculty of Dentistry, University of Malaya (DF PE1103/0037(L)). The study was conducted in accordance with International Conference on Harmonisation Good Clinical Practice (ICH-GCP) guidelines and the Declaration of Helsinki. Subjects were systemically healthy individuals who had at least 12 teeth present (excluding third molars). Subjects, who had received periodontal treatment or antibiotics within the past four months or were pregnant at the time of recruitment were excluded from the study. All examinations on the subjects were conducted by a qualified examiner. Written informed consent was obtained from all subjects. In this study, a total of 90 serum samples were collected, which included 42 healthy controls and 48 patient specimens (collected from 9 mild CP, 12 moderate CP, 19 severe CP (Eke et al., 2012), and 8 AP (Van Der Velden, 2005) patients).

### Samples collection and preparation

Collected samples were divided, classified, and labelled based on the representative stages of periodontitis (i.e., healthy, mild CP, moderate CP, severe CP, and AP). The stages of periodontitis were classified through clinical diagnostic criteria such as PPD and CAL, which was based on the suggested CDC-AAP (Center for Disease Control & Prevention–American Academy of Periodontology) case definitions (Eke et al., 2012). The samples were pooled together by form of periodontitis (10 μl each) into separate microcentrifuge tubes for later use as primary antibodies in the immunoproteomic assay. All samples were stored at a constant temperature of −20 °C for further analysis through 2-DE.

### Two-dimensional electrophoresis (2-DE)

Two-dimensional electrophoresis (2-DE) was performed as previously described (Chen et al., 2014; Chen et al., 2008). Unfractunated whole serum samples (10 μl) were lysed, rehydrated in lysis buffer (2 M thiourea, 8 M urea, 4% CHAPS, 1% dithreitol and 2% pharmalyte), and subjected to isoelectric focusing in 11-cm rehydrated precast Immobiline Drystrips pH 4–7 (GE Healthcare Bioscience, Uppsala, Sweden) overnight, using the Protean Isoelectric Focusing Cell (Biorad Inc., Berkeley, CA, USA). The second-dimensional separation of focused samples in the gel strips was performed through sodium dodecyl sulfate-polyacrylamide gel electrophoresis (SDS-PAGE) using 10% linear polyacrylamide gels. A previously described silver staining method was used to develop the 2-DE gels (Heukeshoven & Dernick, 1988), and a modified silver staining technique was used for mass spectrometry (MS) analysis (Snevechenko et al., 1996). All samples were analysed in duplicate.

### Image and statistical analysis

The ImageQuant™ LAS500 (GE Healthcare Bioscience, Uppsala, Sweden) was used to visualize and store the silver-stained 2-DE gel images. Detection, matching, and quantification of the distinct protein spots were performed using the Image Master 2D Platinum Software, version 7.0 (GE Healthcare Bioscience, Uppsala, Sweden). Normalization was subsequently performed to evaluate proteins that were differentially expressed in the serum by correcting the spot quantification values and gel-to-gel variation unrelated to expression changes. This was achieved by taking into account total densities from the gel images (i.e., raw quantity of each gel spot was divided by the total quantity of all spots within the gel). The percentage of a given protein taken against total spot volume of all proteins (including the unresolved peptides) in the gel was calculated to give a percent of volume contribution (vol%) (Chen et al., 2008). All protein concentration values are presented as means of percentage volume (% volume) ± standard deviations (SD). Statistical analysis was done using the Student’s *t*-test to analyse differences between controls and patients. A p-value of less than 0.05 (p < 0.05) was considered to be statistically significant.

### Mass spectrometry analysis and database search

Prior to MS analysis, spots of interest were excised and subjected to in-gel tryptic digestion using the commercially available Proteo Extract™. All-in-One Trypsin Digestion Kit (Calbiochem, Darmstadt, Germany). MS analysis was then performed at the Faculty of Biological Science Proteomic Centre, National University of Singapore. The digested peptides were first mixed with 1.2 ml of CHCA matrix solution (5 mg/ml of cyano-4-hydroxy-cinnamic acid in 0.1% trifluoroacetic acid (TFA) and 50% acetonitrile (ACN)) and spotted onto MALDI target plates. An ABI 4800 Proteomics Analyzer MALDI-TOF/TOF Mass Spectrometer was used for spectra analysis (Applied Biosystems, CA, USA). The MASCOT search engine (version 2.1; Matrix Science, London, UK) was employed for database searches after MS analysis. In addition, GPS Explorer software (version 3.6; Applied Biosystems, CA, USA) was utilized along with MASCOT to identify peptides and proteins. The applied search parameters allowed for N-terminal acetylation, C-terminal carbamidomethylation of cysteine (fixed modification), and methionine oxidation (variable modification). Furthermore, the peptide and fragment mass tolerance settings were 100 ppm and 60.2 Da, respectively. In addition, the peptide mass fingerprinting (PMF) parameters were set as follows: one missed cleavage allowed in the trypsin digest, monoisotropic mass value, 60.1-Da peptide mass tolerance, and 1+ peptide charge state. During the initial phase, peptides were identified using the ProteinPilot proteomics software on the Mass Spectrometer (Applied Biosystems, Foster City, CA, USA). A score reflecting the relationship between theoretically and experimentally determined masses was calculated and assigned. These analyses were conducted using the International Protein Index (http://www.ebi.ac.uk/IPI) and NCBI Unigene human databases (version 3.38). A score of > 82 was considered to be significant in the MASCOT NCBI database.

### Immunoblotting

The 2-DE analysed sera were grouped into 25 categories based on immunoblotting with primary antibodies (pooled sera) from healthy controls and the four stages of periodontitis (Table 1). The Trans-blot® Turbo™ Transfer Starter System (Biorad Inc., Berkeley, CA, USA) was used to transfer the separated proteins from the 2-DE gels onto nitrocellulose membranes (0.45 μm) (Biorad Inc., Berkeley, CA, USA). Blotted nitrocellulose membranes were then blocked using Superblock (Pierce, Rockford, IL, USA) for 1 h and subsequently incubated overnight (4 °C) with IgM (pooled sera from patients or controls that contain the primary antibodies against various targets) as shown in Table 1. After incubation, membranes were washed three times (5 min each) with TBS-T (Tris-buffered saline Tween-20, pH 7.5). The membranes were then incubated with monoclonal anti-human IgM conjugated to horseradish peroxidase (HRP) (Invitrogen, Carlsbad, CA, USA) at a dilution of 1:6,000 for 1 h at room temperature. Finally, the membranes were developed with chemmiluminescent (ECL) substrate (Pierce Rockford, IL, USA) and visualized with ImageQuant LA500 (GE Healthcare Bioscience, Uppsala, Sweden).

**Table 1:** Incubation of the 2-DE blotted nitrocellulose membranes with sera of patients or controls (as primary antibody) was conducted overnight (4°) prior to monoclonal anti-human IgM-HRP.

Category	Patient sera	Probed primary antibody (pooled sera)
1	Normal	Normal
2	Normal	Mild chronic periodontitis
3	Normal	Moderate chronic periodontitis
4	Normal	Severe chronic periodontitis
5	Normal	Aggressive periodontitis
6	Mild chronic periodontitis	Normal
7	Mild chronic periodontitis	Mild chronic periodontitis
8	Mild chronic periodontitis	Moderate chronic periodontitis
9	Mild chronic periodontitis	Severe chronic periodontitis
10	Mild chronic periodontitis	Aggressive periodontitis
11	Moderate chronic periodontitis	Normal
12	Moderate chronic periodontitis	Mild chronic periodontitis
13	Moderate chronic periodontitis	Moderate chronic periodontitis
14	Moderate chronic periodontitis	Severe chronic periodontitis
15	Moderate chronic periodontitis	Aggressive periodontitis
16	Severe chronic periodontitis	Normal
17	Severe chronic periodontitis	Mild chronic periodontitis
18	Severe chronic periodontitis	Moderate chronic periodontitis
19	Severe chronic periodontitis	Severe chronic periodontitis
20	Severe chronic periodontitis	Aggressive periodontitis
21	Aggressive periodontitis	Normal
22	Aggressive periodontitis	Mild chronic periodontitis
23	Aggressive periodontitis	Moderate chronic periodontitis
24	Aggressive periodontitis	Severe chronic periodontitis
25	Aggressive periodontitis	Aggressive periodontitis

### Functional annotation enrichment and protein interaction analysis

Functional annotation enrichment and protein interactions were performed using web-based bioinformatics tools. DAVID v6.7 (Database for Annotation, Visualization and Integrated Discovery; http://david.abcc.ncifcrf.gov) was used for protein functional enrichment analysis. DAVID v6.7 is a web-based integrated biological knowledgebase and analytical tool that systematically extracts biological meaning from large lists of genes and proteins (Huang, Sherman & Lempicki, 2008). This functional annotation was considered to be significant when a p-value of less than 0.05 (p < 0.05) was obtained. Furthermore, the identified proteins were evaluated using STITCH v4.0 (Search Tool for the Retrieval of Interacting Genes; http://david.abcc.ncifcrf.gov), which is a web-based resource to explore known and predicted interactions between proteins and chemicals (Kuhn et al., 2008). Furthermore, the proteins found to be differently expressed in this study were subjected to Ingenuity Pathway Analysis (IPA), which was used to identify their relationships with other protein networks within the knowledge database through computational algorithms. The highest-scoring networks were analysed to extract relationships between pathways that might link candidate proteins to one another.

## Results and Discussion

### Image analysis of 2-DE serum protein profiling

**Figure 1:**
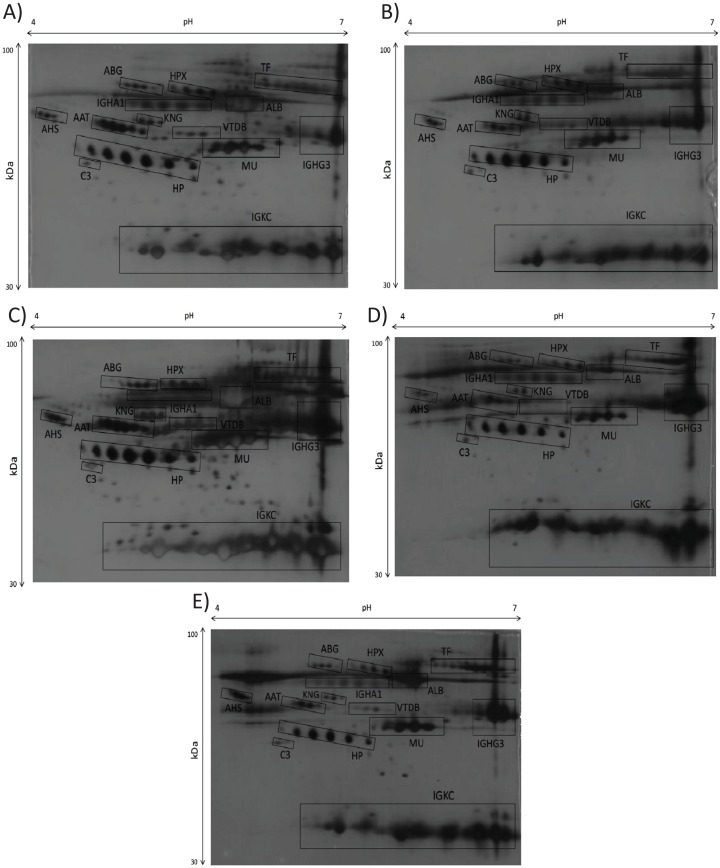
(A) 2-DE serum protein profiles of normal sera, (B) mild/early chronic periodontitis sera, (C) moderate chronic periodontitis sera, (D) severe chronic periodontitis sera, (E) and aggressive periodontitis sera. All serum samples of controls and patients were subjected to 2-DE and silver staining. Protein spot were evaluated and analysed using Image Master Platinum version 7.0. The acidic side of the membranes is to the left and the relative molecular mass declines from the top.

High-resolution serum proteome profiles from normal controls and periodontitis patients (i.e., mild CP, moderate CP, severe CP, and AP) were obtained through 2-DE separation and silver staining of unfractionated serum samples. Fourteen major protein clusters were consistently resolved in all 2-DE silver-stained profiles. Representative 2-DE serum protein profiles from all categories of periodontitis patients and controls are presented in Fig. 1.

### Identification of expressed biomarkers using mass spectrometry

The identity of the 2-DE detected proteins was further verified by subjecting the protein spot clusters to MALDI-TOF/TOF analysis, followed by database searches. The protein clusters were identified as albumin (ALB), α2-HS glycoprotein (AHSG), α1-antitrypsin (A1AT), α1-B glycoprotein (A1BG), Complement C3 (C3), haptoglobin (HP), hemopexin (HPX), Ig alpha-1 chain C region (IGHA1), Ig gamma-3 chain C region (IGHG3), Ig kappa chain C region (IGKC), Ig mu chain C region (IGHM/MU), kininogen (KNG1), transferrin (TRF), and vitamin D-binding protein (VTD). All of the proteins were aberrantly expressed in all 4 categories of periodontitis as compared to normal control. Data regarding protein entry name, protein name, gene name, accession number, nominal mass and MOWSE protein score for each protein are presented in Table 2.

**Table 2:** Mass spectrometric identification of host-specific protein spot clusters from serum protein profiles using the MASCOT search engine and the SWISS-PROT database accessed on 30.11.2014.

Protein entry name	Protein name	Gene name	Accession number	Nominal mass (kDa)	MOWSE protein score
AHSG	α-2-HS glycoprotein	AHSG	P02765	39	63
ALB	Serum albumin	ALB	P02768	69	386
A1AT	α-1-antitrypsin	SERPINA 1	P01009	46	68
A1BG	α-1B glycoprotein	A1BG	P04217	54	107
C3	Complement C3	C3	P01024	187	65
HP	Haptoglobin	HP	P00738	45	98
HPX	Hemopexin	HPX	P02790	51	95
IGHA1	Ig alpha-1 chain C region	IGHA1	P01876	37	61
IGHG3	Ig gamma-3 chain C region	IGHG3	P01860	41	62
IGKC	Ig kappa chain C region	IGKC	P01834	11	69
IGHM/MU	Ig mu chain C region	IGHM	P01871	49	326
KNG1	Kininogen 1	KNG1	P01042	71	66
TF	Serotransferrin	TF	P02787	77	61
VTDB	Vitamin D-binding protein	GC	P02774	52	71

**Figure 2:**
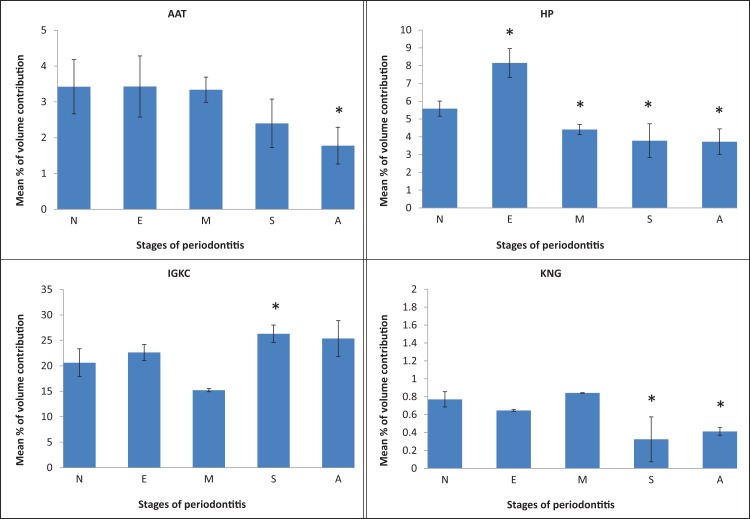
Percentage of volume contribution (vol%) of significantly detected protein clusters in controls (N), and periodontis patient (mild/early chronic periodontitis (E), moderate chronic periodontitis (M), severe chronic periodontitis (S), and aggressive periodontitis (A)). Asterisks (*) indicates significantly differentiated expression (p < 0.05).

We further analysed the average percentage of volume contributions of the 14 serum proteins detected in control and periodontitis samples. Overall, we found that protein levels were comparable in periodontitis patients and controls, with the exception of A1AT, HP, IGKC, and KNG1 (Fig. 2). Notably, KNG1 expression was significantly lower in severe CP and AP patients. On the other hand, A1AT was decreased by two-fold in AP patients, and HP was decreased in moderate CP, severe CP and AP. In fact, the fold changes (down regulation) of these factors in distinct stages of periodontitis were as follows: 0.42, 0.53, 0.52, 0.79, 0.68 and 0.67, respectively (p < 0.05). Interestingly, the level of HP was increased in mild CP patients, whereas IGKC was the only protein significantly increased in severe CP patients (fold up-regulation: 1.46 and 1.28, respectively; p < 0.05). Thus, these aberrantly expressed proteins might represent effective candidate biomarkers for use in the development of future diagnostic tools for periodontitis disease.

### Differently expressed proteins in periodontits patients

The comparative analysis performed in this study revealed down regulation of A1AT, HP, and KNG1 in different categories of periodontitis. The expression of these three proteins was significantly lower in AP, while only HP and KNG1 showed low expression in severe CP. On the other hand, HP was the only protein found to be significantly diminished in moderate CP.

KNG1 belongs to a group of thiol protease inhibitors known as kininogens, which act as inflammatory mediators and cause an increase in vascular permeability to release prostaglandin, another important regulator of inflammation (Iarovaia, 2000). Our data suggest that sera from patients with severe CP and AP physiologically lacked KNG1. This loss of KNG1 from patient sera might be due to the association of kinins (released from kininogens) with tissue kallikrein. In this regard, the release of kinins from kinninogens is triggered by neutrophil elastase and could result from immune recognition of periodontopathogens (Imamura, Potempa & Travis, 2004).

HP is encoded by the *HP* gene in humans (Dobryszycka, 1997; Wassell, 1999). It is commonly considered as an acute-phase reactant and participates in haemoglobin binding in blood (Ebersole et al., 1997; Kapralov et al., 2009). In addition, HP plays a role in host defence towards pathogens (Eaton, Brandt & Lee, 1982). Our analysis of patient sera revealed that HP expression distinctly correlated with the stage of periodontitis. Indeed, HP levels were higher in mild CP patients and gradually decreased with disease progression. The high levels of HP in mild CP may result from initial bacterial pathogenicity in periodontal tissues. In contrast, decreased HP expression in advanced stages of periodontitis could be caused by formation of haemoglobin–haptoglobin complexes that make haemoglobin iron unavailable to bacterial iron-binding proteins (Eaton, Brandt & Lee, 1982). In addition, the correlative expression of HP may reflect the acute phase response of the body (Chen, Lim & Hashim, 2009). Taken together, these findings highlight the potential prognostic value of HP for monitoring the progress of CP.

A1AT is a serine protease inhibitor (Saunders et al., 2012) that contributes to host defence against invading pathogens by inhibiting their proteolytic enzymes (Carrell, 1986; Starkey & Barrett, 1977). Our data reveal that A1AT levels are decreased by two fold in AP patients, which could be due to the capacity of the periodontopathogens to produce proteolytic enzymes that deplete A1AT. Interestingly, our finding is consistent with a previous report on *Porphyromonas gingivalis*, which is present in deep periodontal pockets of periodontitis patients (Carlsson et al., 1984).

IGKC functions as an antigen-binding agent in humans, and therefore selectively interacts with immunogens. IGKC binding can subsequently alter the biological activity of an antigen. In addition, IGKC is involved in complement activation for direct killing of microbes, immune regulation, and disposal of immune complexes (Gottlieb et al., 1970). This might provide a functional explanation for the higher amounts of IGKC seen in severe CP and AP patients.

### Immunogenic protein identification through 2-DE immunoblotting

In order to further investigate the immunoproteomics analyses, 2-DE immunobloting using periodontitis and normal control sera was performed according to the 25 conditions listed in Table 1. The use of normal sera against normal and periodontitis sera was to verify that reactions were restricted to periodontitis. Notably, only IGHG3 was detected in the normal control.

IgM antibodies are secreted by B cells upon stimulation with primary antigen and are important for initial defence mechanisms (Boes et al., 1998). In this regard, IgM is capable of recognizing several pathogens, including lymphocytic choriomeningitis virus (LCMV), *Listeria monocytogenes*, vaccinia virus (vacc-WR), and vesicular stomatitis virus (VSV) (Boes, 2000). Therefore, the identification of immunogenic proteins recognized by IgM in patient sera could allow for the early detection of periodontitis with high specificity and sensitivity.

**Figure 3:**
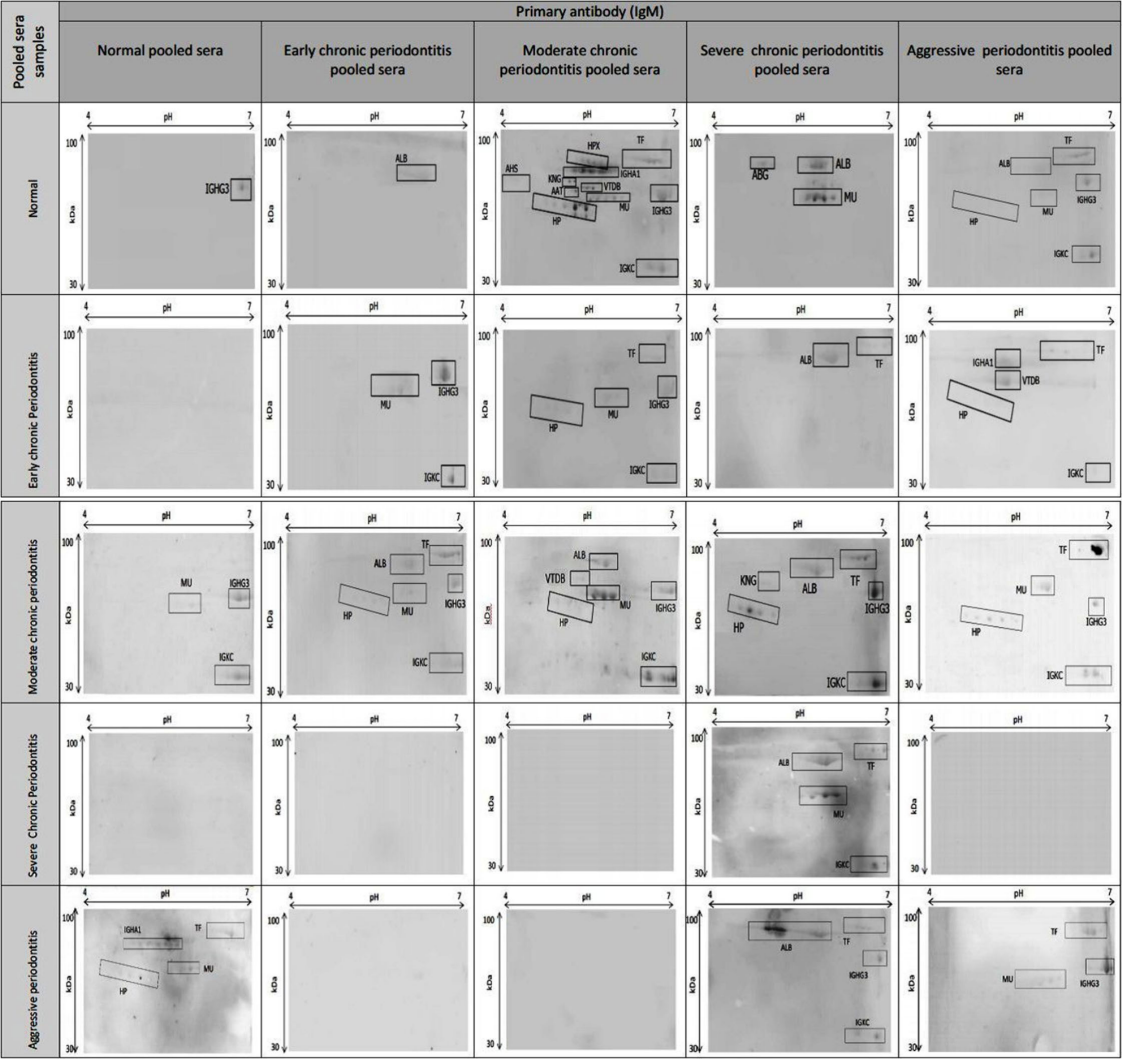
Immunogenic proteins present on the 2-DE immunoblotted nitrocellulose membrane. All serum samples of patients and controls were subjected to 2-DE and blotted onto the nitrocellulose membrane prior to probing with pooled sera and monoclonal anti-human IgM-HRP.

The use of 2-DE immunoblots and MS identification confirmed that all of the observed proteins were host-specific. In addition, it revealed that the immunoblot profiles were distinctive for periodontitis patients and controls, with the exception of categories 6, 16, 17, 18, 20, 22, and 23 (Table 1). The proteins in these categories were not reactive to IgM in the natural condition. Figure 3 illustrates the presence of immunogenic proteins within each category. Based on this immunobloting technique, we found that HP, IGKC, and IGHM of moderate CP were immunoreactive towards the primary antibody (IgM). On the other hand, ALB, HPX, IGHM, and IGKC could be detected in category 19, which corresponded to severe CP serum probed with sever CP IgM.

Furthermore, our immunoproteomic data demonstrated that IgM from severe CP patients was the sole autoantibody capable of detecting host specific immunogenic proteins from all groups of periodontitis and controls (Table 3). Four host proteins (ALB, IGHM, IGKC, and TRF) specifically interacted with the IgM from severe CP and severe CP patient serum. Notably, these factors are known to be involved in bacterial invasion processes (Guevara et al., 2012; Jefferis & Lefranc, 2009; Matsuzaki et al., 1999; Skaar, 2010), further supporting their presence in periodontitis. Also, IgM of severe CP was capable of differentiating periodontitis stages as well as normal controls. This indicates that these proteins could be used as potential biomarkers to diagnose and differentiate the stages of periodontitis, improving the accuracy of current methods.

**Table 3:** Incubation of the 2-DE blotted nitrocellulose membranes with pool sera of SCP patients as primary antibody was conducted overnight at 4° prior to monoclonal anti-human IgM-HRP.

Patients sera	Primary antibody	Immunogenic proteins detected
Normal	SCP pooled sera	ABG, ALB, IGHM
ECP	SCP pooled sera	ALB, TRF
MCP	SCP pooled sera	HP, KNG, ALB, TRF, IGHG3, IGKC
SCP	SCP pooled sera	ALB, TRF, IGHM, IGKC
AP	SCP pooled sera	ALB, TRF, IGHG3, IGKC

Based on the immunoblot profiling analysis, we also observed a unique feature of three host-specific proteins from moderate CP, namely HP, IGHM and IGKC. These proteins presented high immunoreactivity to the IgM, suggesting that they are common proteins recognized by the autoantibody.

### Functional annotation and protein interaction analysis

Functional annotation analysis was performed for our 14 candidate host-specific proteins using DAVID v6.7 and IPA. The associated gene ontology (GO) terms (i.e., biological process, cellular component and molecular function) were annoted based on the database. Additionally, in order to further investigate the signalling pathways of the identified proteins, the Kyoto Encyclopedia of Genes and Genomes (KEGG) database was used to perform pathway annotation and enrichment analysis. Indeed, significant categories could be identified through the expression analysis systemic explorer (EASE) score, which is a modified Fisher Exact p-value (threshold of significance set at p < 0.05). IPA analysis identified three significant networks with scores of 18, 3 and 3, respectively (Table 4). The highest-scoring network, which comprises 14 of the identified proteins in our study, included the following functional processes: inflammatory response, cellular growth, proliferation, and cellular function/maintenance. The remaining two networks showing significant scores involved processes such as cell death and survival, cellular assembly/organization and disease (i.e., cancer, gastrointestinal disease, and hepatic disease).

**Table 4:** Functional annotation analysis of 14 identified proteins using the DAVID bioinformatics resource (DAVID v6.7). (A) Gene Ontology term. Top highest relevant enriched gene ontology terms with EASE score of p < 0.05 (a modified Fisher Exact p-Value) are listed for “Biological Process,” “Cellular Component,” and “Molecular Function,” respectively. (B) KEGG Pathway analysis by DAVID bioinformatics resource. (C) Functional enrichment analysis of 14 identified proteins using Ingenuity Pathway Analysis (IPA) a computational algorithms to identify networks consisting of proteins of interest and their interactions with other proteins in the knowledge database.

(A)						
GO term	Protein count	%	EASE score	Fold enrichment	Benjamini score	Protein ID
**Biological process**						
Defence response	6	46	7.02E-05	10.99	0.010341	AHSG, HP, KNG1, A1AT, C3, TF
Inflammatory response	5	39	9.44E-05	17.34	0.009274	AHSG, KNG1, A1AT, C3, TF
Response to wounding	5	39	6.17E-04	10.63	0.025758	AHSG, KNG1, A1AT, C3, TF
Acute inflammatory response	4	31	5.83E-05	46.01	0.017115	AHSG, A1AT, C3, TF
**Cellular component**						
Extracellular region	12	92	1.42E-09	6.36	5.54E-08	ALB, A1BG, AHSG, VTDB, HP, HPX, IGHG3, IGHM, KNG1, A1AT, C3, I
Extracellular space	9	69	9.34E-09	13.99	1.82E-07	ALB, AHSG, VTDB, HP, HPX, KNG1, A1AT, C3, TF
Extracellular region part	9	69	1.32E-07	9.99	1.72E-06	ALB, AHSG, VTDB, HP, HPX, KNG1, A1AT, C3, TF
**Molecular function**						
Inhibitor activity	4	31	2.11E-04	29.85	0.012572	AHSG, KNG1, A1AT, C3
Peptidase inhibitor activity	4	31	2.47E-04	28.27	0.007385	AHSG, KNG1, A1AT, C3
Enzyme inhibitor activity	4	31	0.001297	16.03	0.019277	AHSG, KNG1, A1AT, C3
Antigen binding	3	23	9.80E-04	57.96	0.019424	IGHA1, IGHG3, IGHM, IGKC

(B)						
Signalling pathway	Protein count	%	EASE score	Fold enrichment	Benjamini score	Protein ID
Complement and coagulation cascades	3	23	1.81E-04	73.69565	3.63E-04	KNG1, A1AT, C3

(C)			
Top network	Score	Focus protein	Protein ID
Inflammatory response, cellular growth and proliferation, cellular function and maintenance	18	9	ABCA1, ABCB1, AGER, AGT, **AHSG**, **ALB**, ANGPTL3, APOA1, APOA2, APOH, ARF6, BMX, **C3**, CANX, CCL4, CEBPB, CFTR, Cg, CLDN2, CPB2, CSH1/CSH2, DEFB103A/DEFB103B, EGFR, ELF3, FGFR2, Fibrinogen, FUT8, G6PC, GFER, HABP2, hemoglobin, HFE, HNF1A, HNF1B, **HP**, **HPX**, Iga, **IGHM**, IL1, ITCH, ITGAM, JAG1, **KNG1**, KRT17, LCN2, MBL2, miR-21-5p (and other miRNAs w/seed AGCUUAU), NFkB (complex), NR1H3, NR1H4, NR5A2, PIAS3, PLC gamma, PLD2, PPAP2B, PTPRS, RHOB, RNF31, SAA, SDC4, **SERPINA1**, TAB2, TAP1, **TF**, TFRC, TLR5, TLR, TLR9, TNFSF13B, WWP2
Cell death and survival, cellular assembly and organization, connective tissue disorders	3	1	FCGR2C, **IGHG3**
Cancer, gastrointestinal disease, hepatic system disease	3	1	ANXA2, **GC**

Analysis of GO biological processes revealed that A1AT, AHSG, C3, KNG1, and TF are involved in host defence, inflammatory response, acute inflammation, and wound responses. Based on the KEGG pathway analysis, we found that A1AT, KNG1 and C3 were also associated with the complement and coagulation cascades. The complement system is a known mediator of innate immunity and constitutes a non-specific defence mechanism against pathogens (Markiewski et al., 2007). Furthermore, IPA analysis revealed that nine proteins identified in our study are associated with the inflammatory response, cellular growth/proliferation, and cellular function/maintenance (Table 4). All of these annotations are consistent with the immediate response of the host to infection or injury in periodontal disease. Therefore, since it is known that periodontal disease is mediated by host defence and the inflammation (Cekici et al., 2014), these findings emphasize the role of these identified proteins in periodontitis.

**Figure 4:**
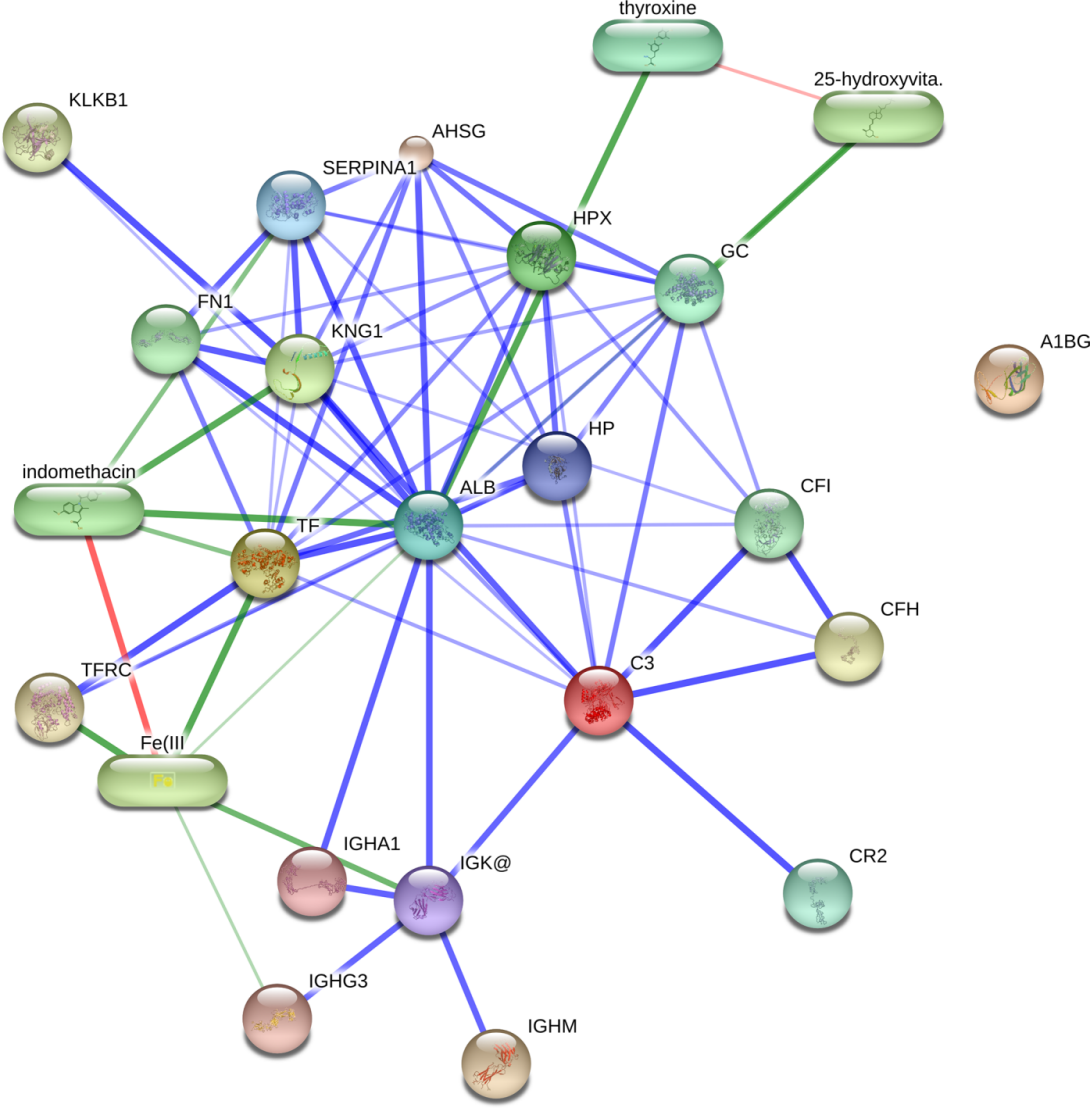
Interaction networks of identified host specific proteins using STITCH v4.0. The interactions are displayed in confidence view. Stronger associations are represented by thicker lines. Protein-proteins interactions are shown in blue, chemical-protein interactions in green and interactions between chemicals are red. Chemical-chemical links are used to extend the network. The findings reveal most of the identified proteins have an established confident link with each other in the interaction network.

In addition, STITCH v4.0 was employed to identify binding partners for the proteins identified in this study and to generate a protein interaction network (Fig. 4). STITCH v4.0 is a web-based resource to explore known and predicted interactions of proteins and chemicals. It integrates widely dispersed information found across numerous databases and the literature (e.g., interaction with metabolic pathways, results from binding experiments and drug-target relationships) to detect known and predicted interactions between proteins and chemicals through genomic context-based inferences and text-mining protocols (Kuhn et al., 2008). Based on this analysis, we identified interactions between the indentified proteins and factors associated with the complement and coagulation pathways (i.e., CF1, CFH, CR2, KLKB1 and FN1). These results further support data obtained from the DAVID and IPA analyses.

## Conclusion

In the present study, we found that HP, KNG1, A1AT and IGKC were differentially expressed in sera collected from periodontal patients. Notably, the identification of these immunogenic proteins in patient sera could contribute to early detection of periodontitis with high specificity and sensitivity. Our findings also revealed that the antibody extracted from patients with severe CP has the most immunogenic properties to classify the distinct stages of periodontitis. Therefore, our results suggest that these differentially expressed host immune response proteins might represent valuable biomarkers for periodontal screening, thereby improving the accuracy of current diagnostic methods to promote early delivery of therapeutic measures against periodontitis. Furthermore, the role of these proteins in host inflammatory and defence responses also highlights their importance as potential biomarkers for periodontal disease. Further study within clinically representative populations will be needed to validate these promising findings.

## Additional Information and Declarations

### Data Deposition

The following information was supplied regarding data availability: MASCOT search engine and SWISS-PROT database. Accession numbers P02765, P02768, P01009, P04217, P01024, P00738, P02790, P01876, P01860, P01834, P01871, P01042, P02787, P02774.

## References

[ref-1] Albandar JM, Brunelle JM, Kingman A (1999). Destructive periodontal disease in adults 30 years of age and older in the United States, 1988–1994. Journal of Periodontology.

[ref-2] Armitage GC (1999). Development of a classification system for periodontal diseases and conditions. Annals of Periodontology.

[ref-3] Avrameas S (1991). Natural autoantibodies: from ‘horror autotoxicus’ to ‘gnothi seauton’. Immunology Today.

[ref-4] Bartold PM, Van Dyke TE (2013). Periodontitis: a host-mediated disruption of microbial homeostasis. Unlearning learned concepts. Periodontology 2000.

[ref-5] Boes M (2000). Role of natural and immune IgM antibodies in immune responses. Molecular Immunology.

[ref-6] Boes M, Prodeus AP, Schmidt T, Carroll MC, Chen J (1998). A critical role of natural immunoglobulin M in immediate defense against systemic bacterial infection. Journal of Experimental Medicine.

[ref-7] Carlsson J, Herrmann BF, Höfling JF, Sundqvist GK (1984). Degradation of the human proteinase inhibitors alpha-1-antitrypsin and alpha-2-macroglobulin by Bacteroides gingivalis. Infection and Immunity.

[ref-8] Carrell RW (1986). Alpha 1-Antitrypsin: molecular pathology, leukocytes, and tissue damage. Journal of Clinical Investigation.

[ref-9] Casali P, Schettino EW (1996). Structure and function of natural antibodies. Immunology of Silicones.

[ref-10] Cekici A, Kantarci A, Hasturk H, Van Dyke TE (2014). Inflammatory and immune pathways in the pathogenesis of periodontal disease. Periodontology 2000.

[ref-11] Chapple ILC (1997). Periodontal disease diagnosis: current status and future developments. Journal of Dentistry.

[ref-12] Chen Y, Azman SN, Kerishnan JP, Zain RB, Chen YN, Wong YL, Gopinath SCB (2014). Identification of host-immune response protein candidates in the sera of human oral squamous cell carcinoma patients. PLoS ONE.

[ref-13] Chen Y, Lim B-K, Hashim OH (2009). Different altered stage correlative expression of high abundance acute-phase proteins in sera of patients with epithelial ovarian carcinoma. Journal of Hematology & Oncology.

[ref-14] Chen Y, Lim B-K, Peh S-C, Abdul-Rahman PS, Hashim OH (2008). Profiling of serum and tissue high abundance acute-phase proteins of patients with epithelial and germ line ovarian carcinoma. Proteome Science.

[ref-15] Coutinho A, Kazatchkine MD, Avrameas S (1995). Natural autoantibodies. Current Opinion in Immunology.

[ref-16] Dobryszycka W (1997). Biological functions of haptoglobin-new pieces to an old puzzle. European Journal of Clinical Chemistry and Clinical Biochemistry.

[ref-17] Eaton JW, Brandt P, Lee J (1982). Haptoglobin: a natural bacteriostat. Science.

[ref-18] Ebersole JL, Machen RL, Steffen MJ, Willmann DE (1997). Systemic acute-phase reactants, C-reactive protein and haptoglobin, in adult periodontitis. Clinical & Experimental Immunology.

[ref-19] Eke PI, Dye BA, Wei L, Thornton-Evans GO, Genco RJ (2012). Prevalence of periodontitis in adults in the United States: 2009 and 2010. Journal of Dental Research.

[ref-20] Fearon DT, Locksley RM (1996). The instructive role of innate immunity in the acquired immune response. Science.

[ref-21] Gottlieb PD, Cunningham BA, Rutishauser US, Edelman GM (1970). Covalent structure of a human γG-immunoglobulin. VI. Amino acid sequence of the light chain. Biochemistry.

[ref-22] Guevara M, Terra C, Nazar A, Solà E, Fernández J, Pavesi M, Arroyo V, Ginès P (2012). Albumin for bacterial infections other than spontaneous bacterial peritonitis in cirrhosis. A randomized, controlled study. Journal of Hepatology.

[ref-23] Hatem AE (2012). Epidemiology and Risk Factors of Periodontal Disease.

[ref-24] Heukeshoven J, Dernick R (1988). Improved silver staining procedure for fast staining in PhastSystem development unit. I. Staining of sodium dodecyl sulfate gels. Electrophoresis.

[ref-25] Huang DW, Sherman BT, Lempicki RA (2008). Systematic and integrative analysis of large gene lists using DAVID bioinformatics resources. Nature Protocols.

[ref-26] Iarovaia G (2000). Kallikrein-kinin system: novel facts and concepts (literature review). Voprosy Meditsinskoi Khimii.

[ref-27] Imamura T, Potempa J, Travis J (2004). Activation of the kallikrein-kinin system and release of new kinins through alternative cleavage of kininogens by microbial and human cell proteinases. Biological Chemistry.

[ref-28] Jefferis R, Lefranc M-P (2009). Human immunoglobulin allotypes: possible implications for immunogenicity. MAbs.

[ref-29] Kapralov A, Vlasova II, Feng W, Maeda A, Walson K, Tyurin VA, Huang Z, Aneja RK, Carcillo J, Bayır H, Kagan VE (2009). Peroxidase activity of hemoglobin haptoglobin complexes: covalent aggregation and oxidative stress in plasma and macrophages. Journal of Biological Chemistry.

[ref-30] Kinney JS, Ramseier CA, Giannobile WV (2007). Oral fluid–based biomarkers of alveolar bone loss in periodontitis. Annals of the New York Academy of Sciences.

[ref-31] Kuhn M, von Mering C, Campillos M, Jensen LJ, Bork P (2008). STITCH: interaction networks of chemicals and proteins. Nucleic Acids Research.

[ref-32] Lin H-S, Talwar HS, Tarca AL, Ionan A, Chatterjee M, Ye B, Wojciechowski J, Mohapatra S, Basson MD, Yoo GH, Peshek B, Lonardo F, Pan C-JG, Folbe AJ, Draghici S, Abrams J, Tainsky MA (2007). Autoantibody approach for serum-based detection of head and neck cancer. Cancer Epidemiology Biomarkers & Prevention.

[ref-33] Löe H, Theilade E, Jensen SB (1965). Experimental gingivitis in man. Journal of Periodontology.

[ref-34] Markiewski MM, Nilsson B, Ekdahl KN, Mollnes TE, Lambris JD (2007). Complement and coagulation: strangers or partners in crime?. Trends in Immunology.

[ref-35] Matsuzaki G, Vordermeier HM, Hashimoto A, Nomoto K, Ivanyi J (1999). The role of B cells in the establishment of T cell response in mice infected with an intracellular bacteria, *Listeria monocytogenes*. Cellular Immunology.

[ref-36] Pereira P, Forni L, Larsson EL, Cooper M, Heusser C, Coutinho A (1986). Autonomous activation of B and T cells in antigen-free mice. European Journal of Immunology.

[ref-37] Preshaw PM (2009). Definitions of periodontal disease in research. Journal of Clinical Periodontology.

[ref-38] Ramfjord SP (1959). Indices for prevalence and incidence of periodontal disease. Journal of Periodontology.

[ref-39] Saunders DN, Tindall EA, Shearer RF, Roberson J, Decker A, Wilson JA, Hayes VM (2012). A novel SERPINA1 mutation causing serum alpha1-antitrypsin deficiency. PLoS ONE.

[ref-40] Skaar EP (2010). The battle for iron between bacterial pathogens and their vertebrate hosts. PLoS Pathogens.

[ref-41] Snevechenko A, Wilm M, Vorm O, Mann M (1996). Mass spectrometric sequencing of proteins silver-stained polyacrilamide gels. Analytical Chemistry.

[ref-42] Starkey PM, Barrett A, Barrett AJ (1977). α2-Macroglobulin, a physiological regulator of proteinase activity. Proteinases in Mammalian Cells and Tissues.

[ref-43] Susin C, Haas AN, Albandar JM (2014). Epidemiology and demographics of aggressive periodontitis. Periodontology 2000.

[ref-44] Tlaskalová-Hogenová H, Mandel L, Stĕpánková R, Bártová J, Barot R, Leclerc M, Kovárů F, Trebichavský I (1991). Autoimmunity: from physiology to pathology. Natural antibodies, mucosal immunity and development of B cell repertoire. Folia Biologica.

[ref-45] Vaithilingam R, Safii S, Baharuddin N, Karen-Ng LP, Saub R, Ariffin F, Ramli H, Sharifuddin A, Hidayat MFH, Raman R, Chan YK, Rani NA, Rahim RA, Shahruddin N, Cheong SC, Bartold PM, Zain RB (2015). Establishing and managing a periodontal biobank for research: the sharing of experience. Oral Diseases.

[ref-46] Van Der Velden U (2005). Purpose and problems of periodontal disease classification. Periodontology 2000.

[ref-47] Vollmers HP, Brändlein S (2006). Natural IgM antibodies: the orphaned molecules in immune surveillance. Advanced Drug Delivery Reviews.

[ref-48] Wassell J (1999). Haptoglobin: function and polymorphism. Clinical Laboratory.

[ref-49] Wiebe CB, Putnins EE (2000). The periodontal disease classification system of the American Academy of Periodontology–an update. Journal of Canadian Dental Association.

